# Genomic variation in myeloma: design, content, and initial application of the Bank On A Cure SNP Panel to detect associations with progression-free survival

**DOI:** 10.1186/1741-7015-6-26

**Published:** 2008-09-08

**Authors:** Brian Van Ness, Christine Ramos, Majda Haznadar, Antje Hoering, Jeff Haessler, John Crowley, Susanna Jacobus, Martin Oken, Vincent Rajkumar, Philip Greipp, Bart Barlogie, Brian Durie, Michael Katz, Gowtham Atluri, Gang Fang, Rohit Gupta, Michael Steinbach, Vipin Kumar, Richard Mushlin, David Johnson, Gareth Morgan

**Affiliations:** 1 Cancer Center, University of Minnesota, Minneapolis, MN, USA; 2 Cancer Research and Biostatistics, Seattle, WA, USA; 3 Dana Farber Cancer Institute, Boston, MA, USA; 4 North Memorial Hospital, Minneapolis, MN, USA; 5 Hematology, Mayo Clinic, Rochester, MN, USA; 6 University of Arkansas Medical Sciences Center, Little Rock, AK, USA; 7 Cedar Sinai Medical Center, Los Angeles, CA, USA; 8 International Myeloma Foundation, Hollywood, CA, USA; 9 Electrical Engineering & Computer Science, University of Minnesota, Minneapolis, MN, USA; 10 IBM Research, TJ Watson Research Center, Yorktown Heights, NY, USA; 11 Royal Marsden Hospital, London, UK

## Abstract

**Background:**

We have engaged in an international program designated the *Bank On A Cure*, which has established DNA banks from multiple cooperative and institutional clinical trials, and a platform for examining the association of genetic variations with disease risk and outcomes in multiple myeloma.

We describe the development and content of a novel custom SNP panel that contains 3404 SNPs in 983 genes, representing cellular functions and pathways that may influence disease severity at diagnosis, toxicity, progression or other treatment outcomes. A systematic search of national databases was used to identify non-synonymous coding SNPs and SNPs within transcriptional regulatory regions. To explore SNP associations with PFS we compared SNP profiles of short term (less than 1 year, *n *= 70) versus long term progression-free survivors (greater than 3 years, *n *= 73) in two phase III clinical trials.

**Results:**

Quality controls were established, demonstrating an accurate and robust screening panel for genetic variations, and some initial racial comparisons of allelic variation were done. A variety of analytical approaches, including machine learning tools for data mining and recursive partitioning analyses, demonstrated predictive value of the SNP panel in survival. While the entire SNP panel showed genotype predictive association with PFS, some SNP subsets were identified within drug response, cellular signaling and cell cycle genes.

**Conclusion:**

A targeted gene approach was undertaken to develop an SNP panel that can test for associations with clinical outcomes in myeloma. The initial analysis provided some predictive power, demonstrating that genetic variations in the myeloma patient population may influence PFS.

## Background

The draft sequence of the human genome published in 2001 [1,2], followed by the more recent improved sequence release of the International Human Genome Consortium [3], have shown that there are large genetic variations in the human genome (polymorphisms). Unlike somatic mutations, polymorphisms are stable and heritable. Polymorphisms include single nucleotide polymorphisms (SNPs), and micro- and minisatellites, and may include heritable insertions and deletions (indels). Significantly, SNPs account for over 90% of genetic variation in the human genome [2]. An important principle that has emerged from the consideration of genetic variation is that disease risk and clinical outcomes can be influenced by individual genetic backgrounds. Thus, while many diseases may have their unique genetic signatures, individual patient outcomes are dependent on heritable variations in a wide variety of genes and pathways affecting cellular functions and drug responses. Moreover, genetic variations in such global functions as inflammation, immunity and cellular signaling in the tumor microenvironment can have an impact on diverse clinical responses.

Multiple myeloma (MM) is a universally fatal disease characterized by the accumulation of malignant plasma cells in the bone marrow [4]. It accounts for 2% of all cancer deaths and 15% of all hematologic malignancies, with about 13,000 deaths per year in the USA [4]. While there are certain common clinical features such as anemia, bone lesions, hypercalcemia, immunodeficiency and renal failure, the disease shows significant heterogeneity with regard to morphology, disease progression, response to therapy and incidence of secondary malignancies. This heterogeneity likely is due, in part, to differences in genetic abnormalities within the malignant clone, as shown in many studies on chromosomal abnormalities [5] and gene expression profiles [6-8].

The growth of MM plasma cells is dependent on a complex interplay among various growth factors, adhesion molecules and other factors in the tumor microenvironment. Thus it might be expected that genetic variations in this interplay could have a profound influence on disease initiation, progression, associated bone complications, and response. Moreover, genetic variation in immunity and inflammation is an important consideration, as are variations in genes coding for drug metabolism and transport. Indeed, death from MM commonly results from infections associated with a severely compromised immune system resulting, in part, from therapeutic toxicities that may be related to variable rates of drug metabolism [9].

In order to address these issues we have engaged in an international program designated as the *Bank On A Cure *(BOAC). A cooperative program was established to bank DNA from multiple cooperative groups and institutional trials, and to develop a platform for examining the association of genetic variation with disease risk and outcomes. BOAC receives samples through Material Transfer Agreements, and clinical outcomes are provided through agreements with the Cancer Research and Biostatistics Group (Seattle) and the University of Minnesota (with Institutional Review Board, IRB, approval). Currently, the bank has over 2100 samples from the USA, representing six different clinical trials, patient-provided BOAC buccal cell kit samples, and unaffected controls accumulated since 1987. In this report we describe the development of a novel custom SNP panel based on the Affymetrix/Gene Chip Targeted Genotyping Platform, which contains 3404 SNPs representing variations in a variety of cellular functions and networks, and its initial application to myeloma DNA samples collected in the BOAC bank. We examined population frequencies in affected and unaffected individuals among different ethnic groups, and we developed some novel early approaches in using the SNP panel to determine whether genomic variations in the patient population influence survival.

## Methods

### Control and patient samples

DNA was prepared from 102 Coriell cell lines [10], representing 31 Caucasian, 24 African American, 23 Hispanic, and 24 Asian racial groups (unaffected by myeloma). DNA samples were also prepared from 143 myeloma patients enrolled in phase III clinical trials: E9486, *n *= 52 [11] and S9321, *n *= 91 [12], with informed consent; and 34 unaffected, spousal controls (all Caucasian). E9486 patients ranging in age from 55 to 70 years were treated with Vincristine, Busulfan, Melphalan, Cyclophossphamide, Prednisone (VBMCP) followed by randomization to no further treatment, IFN-a, or cylcophosphamide; and, although there was variation in survival among all patients, no significant differences in survival were noted among the three arms of the trial [11]. Patients included in this study from S9321 were in the same age range, and received Vincristine, Adriamycin, Dexamethasone (VAD) induction followed by VBMCP [12]. S9321 patients in the trial arm randomized to high dose melphalan+TBI followed by transplant were not included. Patients for this analysis were selected based on progression-free survival (PFS) of less than 1 year (*n *= 70) or greater than 3 years (*n *= 73).

### Custom BOAC SNP chip design and content

**Figure 1:**
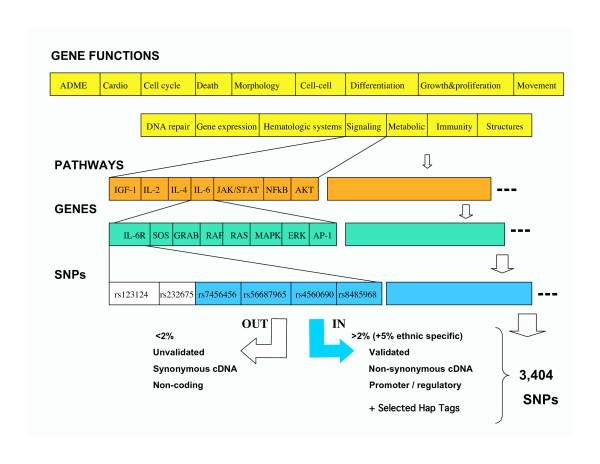
**SNP selection strategy for the BOAC SNP panel**. For full description, see *Methods *and *Results*. Numbers under the cell functions indicate the final number of SNPs on the chip in each category.

A directed, custom BOAC SNP chip design was developed with specific criteria from public and commercial databases. Rather than a total genome wide screen, a plan was undertaken to develop a custom SNP chip, focusing on functionally relevant polymorphisms playing a role in normal and abnormal cellular functions, inflammation and immunity, as well as drug responses. Candidate gene lists were created and each gene in the candidate list was systematically investigated with a selection of SNP databases to harvest SNPs that may have a functional effect on gene action. Figure 1 outlines the approach. Searches for genes were developed, using public and commercial software programs in PubMed, iHOP [13], as well as pathway databases, such as PharmGKB Pathways [14], BioCarta [15], KEGG [16], Ingenuity, and Pathway Assist (Stratagene, Inc.).

The Human Gene Mutation Database [17] contains a searchable database of polymorphisms associated with diseases cited in the literature. This database was used in conjunction with SNP500, SNPper, and MutDB to obtain the SNP id (rs number) of polymorphisms in the gene lists. A systematic search for all non-synonymous SNPs (ie, resulting in coding change) with a validated, minor allele frequency greater than 2% in all of the candidate genes was completed using SNP 500, dbSNP, and Affymetrix databases. SNPs failing to meet a 2% population frequency were included if the frequency was higher than 5% in selected racial subgroups (eg, Asian, African American, Caucasian).

A systematic search of the promoter regions in all the candidate genes for SNPs present in homologous regions between human and mouse with a minor allele frequency greater than 2% were identified using the PromoLign Database [18]. Many of the SNPs selected in this method were seen to lie in or adjacent to transcription binding sites. Some additional promoter SNPs that may affect transcription binding sites were also identified using the FESD [19] database. Affymetrix provided several in-house validated SNP lists, including: inflammation and immunity, drug metabolism, and cancer lists. Three groups of admixture SNPs, which differ in frequency between Asian, African and European groups, were added to allow corrections in data analyses for racial specific variations [20]. TagSNPs in genes influencing drug metabolism and transport were added from the supplementary data from Ahmadi et al. [21]. The full SNP panel includes 3404 SNPs in 983 genes.

Genotyping was performed using the Affymetrix^® ^GeneChip^® ^Scanner 3000 Targeted Genotyping System (GCS 3000 TG System), which utilizes molecular inversion probes to simultaneously identify the 3404 pre-selected SNPs. The protocol has previously been described [22]. All genotyping experiments were performed in strict adherence to the manufacturer's protocol.

### Statistical methods

Patients from the Eastern Cooperative Oncology Group (ECOG) and Southwest Oncology Group (SWOG) trials were selected using the following criteria: they were all Caucasian and between 55 and 70 years of age at diagnosis; patients with IgA subtype were excluded (as this is an independent, poor prognosis variable). Patients with the longest progression-free survival (PFS > 3 years) and patients with the shortest progression-free survival (PFS < 1 year) were selected.

Two approaches were used to determine whether there was true discrimination of SNP genotypes in the PFS analysis, when analyzed as a conglomerate data set.

1) *Leave-one-out cross-validation *[23]. In this approach, the original data set of 143 patients was divided into two groups: one consisting of a single patient and one consisting of the remaining 142 patients. A classification model was built using the 142 patients as a training set and then this classification model was used to classify the single 'left out' patient. For this study, as well as the class label study below, we used a support vector machine (SVM) classifier, as implemented by the Weka package [24], and specified a liner kernel.

2) *Randomization of class labels *[25]. For the original data and labels, we followed the standard practice for building and evaluating a classifier [25], that is, compare the performance of a classifier using the original and randomly shuffled class labels (permutations). There were 143 subjects, consisting of 73 cases and 70 controls. The training set was created by randomly selecting 50 cases and 50 controls and using the remaining subjects as a test set. One hundred runs were performed for the original data and class labels.

We also analyzed each clinical trial data set separately and used the other clinical trial data set as a validation set. Fisher's exact test was used as a univariate screening tool to rank the SNPs by how strongly they are associated with PFS. The top 50 SNPs of each trial with the smallest *p*-value were selected and used in a recursive partitioning analysis. For this recursive partitioning analysis we used RPART from the R software package, a language and environment for statistical computing. The tree-based library RPART was developed as described [26]. The regression tree resulting from the analysis was subsequently pruned in order to avoid over-fitting. This regression tree was used on both the trial it was developed on as well as the other trial for validation purposes. Specificity and sensitivity were determined for each data set.

Finally, we attempted univariate ranking and recursive partitioning of the conglomerate data set (both trials combined) using random forests [27]. Validation was examined by randomly mixing survival data sets and determining and comparing the predictive accuracy of true survival subsets and random subsets.

## Results

### SNP chip panel design

A final custom SNP chip panel of 3404 SNPs from 983 genes meeting the above criteria was produced for the Affymetrix/Gene Chip Targeted Genotyping Platform. A full documented list of SNPs is found in [28] and includes rs assignments, gene identifiers (Entrez), functional grouping, and SNP effect (coding, non-coding, regulatory, haptag). Table 1 summarizes a variety of functional categories represented on the chip. Figure 1S (found in [28]) shows the chromosomal distribution of the SNPs included. Although the SNP chip was not designed to serve as a genome linkage panel, the chromosomal distribution is quite broad (see additional File 1), and may provide functional targets for higher density linkage chips or regions identified by other approaches such as comparative genomic hybridizations or genome wide screens. We also examined representation among a variety of defined metabolic and signaling pathways. Because of the filter criteria, it was found that most pathways did not have a high degree of representation, suggesting SNPs for many of the genes not included may not have coding or regulatory impact. This becomes an important consideration in attempts to associate outcomes with specific pathways. Instead, common functional groupings turned out to be a better analytical target (see below).

**Table 1:** Functional categories on the SNP panel

**Functional Category**	**#Genes**	**#SNPs**
ADME/DMET	130	445
Cancer	406	1558
Carbohydrate Metabolism	69	384
Cell Cycle	230	867
Cell Death	433	1662
Cell Signaling	90	352
Cell-To-Cell Signaling and Interaction	248	880
Cellular Growth and Proliferation	420	1451
Cellular Movement	227	923
DNA Replication, Recombination, and Repair	204	854
Drug Metabolism	20	114
Gene Expression	240	951
Hematological Disease	223	876
Immune Response	247	985
Lipid Metabolism	146	664
Molecular Transport	170	708
Nucleic Acid Metabolism	30	161
Skeletal and Muscular Disorders	64	289
Skeletal and Muscular System Development and Function	77	278
Signaling Kinase, Phosphatase, Transferase	198	885
Inflammation & Immunity	196	813

It is noteworthy to compare the content of the BOAC SNP chip to the SNPs represented on the Affymetrix 500K Array genome wide scan. The 500K Array panel is primarily derived from two restriction enzyme cleavage fragmentations, with SNP representation for each fragment, providing a comprehensive, global SNP panel. Well over 95% of the panel is intragenic, non-coding; and thus, its primarily use is to identify copy number, chromosomal regions, and linkage. Indeed, of the 3404 SNPs on the BOAC SNP chip, only 401 are present on the 500K Array panel. Thus, while the BOAC SNP chip does not have gene wide coverage, it does have a higher density of coding and regulatory content.

### Samples and quality control assessments

For this study, a total of 279 DNA samples were profiled by the BOAC SNP chip. One hundred and thirty-six unaffected controls from the Coriell panel and spouses of myeloma patients were profiled. The Coriell panel included 31 Caucasian, 24 Asian, 23 Hispanic, and 24 African American samples of unaffected individuals. One hundred and forty-three myeloma samples were profiled, from the phase III clinical trials, ECOG E9486 (*n *= 52) and the chemotherapy arm of the ECOG-SWOG intergroup trial S9321 (*n *= 91). Treatment protocols are given in the *Methods *section. This study was in compliance with the Helsinki Declaration, and approved by the IRB at the University of Minnesota (approval # 0311M53428), with patient consents collected by the clinical cooperative groups' trial offices. Among all samples profiled, we had an average SNP call rate of 96%. The profiles of the Coriell panel allowed us to determine allelic frequencies in racial groups and unaffected populations. Of the 3404 SNPs on the BOAC panel, 786 were contained in the SNP500 cancer database, allowing us to determine concordance between the two Coriell data sets. We found very good agreement between our data set and the national database, with an average of > 97% concordance. We also duplicated the profile of a number of samples (*n *= 10), and found better than 99.7% reproducibility between duplicate samples. This concordance and duplication rate was also equivalent when comparing the BOAC SNP panel run in the USA and UK facilities, providing a cross validation between BOAC laboratory sites. Finally, for every batch run of 24 samples, the Affymetrix platform includes a control DNA sample, and this provided continuous monitoring and quality assurance across the study.

### Allelic variations by race

**Figure 2:**
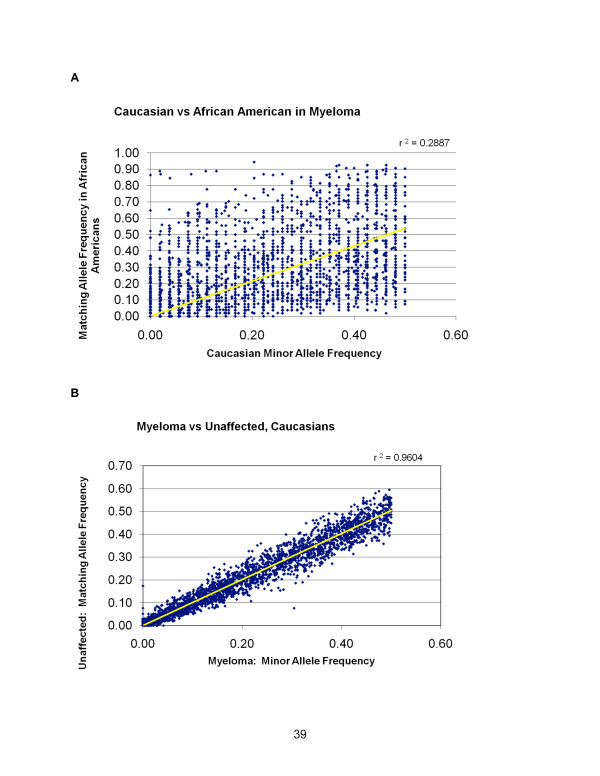
**Racial allelic frequency patterns**. A) Diagonal plot comparing minor allele frequencies between BOAC SNPs of Caucasian versus African American myeloma patients. Note high rate of allelic variation. B) Diagonal plot comparing minor allele frequencies between BOAC SNPs of Caucasian myeloma patients versus unaffected Caucasians.

It has been well established that there are significant allelic frequency differences by race, or ethnic and regional origins [29]. Part of the SNP panel design included the admixture SNP panel that shows significant racial variation. Figure 2A shows a diagonal plot in which each SNP minor allelic frequency is plotted by frequency in the Caucasian (*n *= 92) versus the African American (*n *= 27) myeloma populations. Equivalent frequencies would be expected to cluster on the 45 degree angle; and it is readily apparent that frequencies of many of the SNPs vary widely between races. Indeed, the racial disparities in allelic frequencies were far more significant than could be assessed in case-control or outcome studies, so that subsequent initial survival analyses were done only on a single racial group. Moreover, given the inclusion criteria of SNPs (included if greater than 5% in one racial group), it is noteworthy that 401 SNPs show allelic variation only in the African Americans (ie, no variations seen in Caucasian). In contrast, in a comparison of unaffected samples with affected samples, restricted to Caucasians, there is high concordance across the total panel of SNPs (Figure 2B). This provides an opportunity to examine smaller clusters or functional associations with disease that may be masked by the larger multi-racial pools. However, the object of this study was not to compare variations within different ethnic patient populations.

### Allelic variations associated with progression-free survival

Although genetic deregulation within the tumor population has been shown to stratify clinical outcomes [5-8], a significant impact on therapeutic outcomes may result from genetic variations in germline DNA affecting a number of important functions, including drug metabolism, transport, DNA repair, immune response, growth factors, angiogenesis, etc. To explore the SNP associations on the BOAC SNP chip we chose to examine an extreme phenotype comparison in two phase III clinical trials with similar chemotherapeutic treatments. E9486 patients ranging in age from 55 to 70 years were treated with VBMCP followed by randomization to no further treatment, IFN-a, or cylcophosphamide; and, although there was variation in survival among all patients, no significant differences in survival were noted among the three arms of the trial [11]. Patients included in this study from S9321 were in the same age range, and received VAD induction followed by VBMCP [12]. S9321 patients in the trial arm receiving high dose melphalan+TBI, going on to transplant were not included. The goal was to identify SNPs that may distinguish short term (less than 1 year) versus long term progression-free survivors (greater than 3 years).

While our banking represents one of the largest collections of myeloma specimens, one of the difficulties encountered in this data analysis still results from relatively small sample sizes of patients with similar treatment protocols. With the data sets we had, we used a variety of approaches to determine whether there was true discrimination of SNP associations between the two PFS groups. The 'leave-one-out' approach is a standard approach in classification [23], but is not typically used, due to computational cost, when data sets are large. However, in this case, only 143 classification runs were necessary. The results of those runs performed using an SVM classifier from Weka [24] are summarized in Table 2. SVM is a supervised method used for classification and regression. It belongs to a family of generalized linear classifiers. A special property of SVM is that it simultaneously minimizes the empirical classification error and maximizes the predictive separation.

**Table 2:** Predicted vs. actual survival classes for patients.

	Actual Patient PFS < 1 year	Actual Patient PFS > 3 year
Predicted Patient PFS < 1 year	45	23
Predicted Patient PFS > 3 years	25	50
TOTALS	70	73

The classification accuracy of the leave-one-out approach is (50+45)/143 = 0.66. If there was no true discriminating signal in the data, then the classifiers built by the leave-one-out procedure should produce a table with a relatively evenly distributed number of entries among the four cells, since the classes are of roughly the same size and the predictions should be random. However, the observed table is far from that random distribution. By using Fisher's exact test it is possible to compute the probability (*p*-value) for obtaining a table with the same or better accuracy of prediction by random chance. Specifically, the *p*-value is 7.7 × 10^-5^, which strongly indicates that the result is not due to random chance. The calculated odds ratio (OR) for survival is 3.9 CI (2.0, 7.8). We subsequently focused on SNP subsets that might provide more directed functional associations and found that the best predictor of survival was achieved when just the non-synonymous SNPs and the promolign SNP subset in introns was used. The accuracy of prediction increased to 75.5% OR = 9.6 CI (4.5, 20.5).

**Figure 3:**
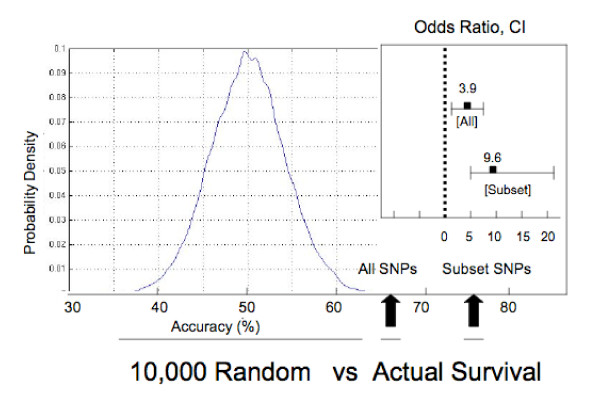
**Survival prediction accuracy versus distribution of random subsets from the BOAC SNP panel**. The 173 SNP profiles were randomly paired 10,000 times, and the accuracy of the SNP prediction was determined, resulting in a distribution of accuracy, centered around 50%. This is compared with survival group prediction accuracy of the full SNP panel (66%) and the subset of SNPs (76%) described in the text. Odds ratios and confidence intervals are given for each. In both cases the *p*-value for predictive power is less than 0.0001.

To determine whether genotypes in the SNP panel had true discriminatory power we randomly permuted the outcome across the two groups and calculated the classification accuracy. A total of 10,000 random group comparisons were performed (ie, survival groups were randomly mixed) with the distribution of accuracy shown in Figure 3. As expected, the most common accuracy was close to 50%, with a random distribution around the mean. Notably, no random grouping achieved the accuracy of the original survival classification of 66%, nor the 75% subset, indicating that, as a group, the SNPs are providing a measure of true discrimination of survival.

Another approach that is commonly used for classification is the generation of random subsets for training and validation. A classification model is built on the training subset, and is evaluated on a separate test set. The process is repeated and yields a distribution of accuracy on the data set labels (eg, short versus long PFS). This is then repeated with random shuffling of the survival data sets to determine whether there is true signal accuracy. For this analysis, we again used a support vector machine classifier, as implemented by the Weka package [25], using a preset linear kernel option). The training set consisted of repeated samples of 50 short term and 50 long term survivors, with the remaining patient samples used for test validation. One hundred runs were performed for the short and long term classifiers, and the average accuracy on test set analysis was 61.4% +/-7.1%. One hundred runs of random mixed set comparisons generated an average accuracy of 47.5% +/-7.3%. This further suggests that there are true differences in the genotypes that impact survival classification. A t-test was performed to evaluate the difference between the classification results based on the original and randomized class labels, resulting in a *p*-value for survival classification of less than 0.0001. This further indicates that, as a group, the SNPs are providing a measure of true discrimination of survival.

Each of the above approaches demonstrated that the SNP panel provided discrimination; however, we attempted to explore possible subsets of SNPs that may drive the association. We used Fisher's exact test as a univariate screening tool to determine the association of SNPs with each trial separately (Tables 3 and 4), then using the top 50 rank ordered SNPs, performed recursive partitioning to identify the combination of SNPs that best distinguish PFS groups. In recursive partitioning each genotype is evaluated on its ability to make a correct prediction, creating a decision node. A pruned decision tree is created in which the minimum number of the strongest nodes creates a group prediction. From the results of each trial, we then validated on the other trial.

**Table 3:** Top SNPs ranked by univariate analysis for trial S9321

**Rank**	**rs ID**	**pval**	**Gene Sym**	**Gene Name**	**SNP Function**
1.0	rs2066534	0.001280	FMO3	Flavin containing monooxygenase 3	intron
2.0	rs696217	0.001877	GHRL	Ghrelin precursor	coding-nonsynon
3.0	rs1043424	0.002404	PINK1	PTEN induced putative Kinase 1	coding-nonsynon
4.0	rs174680	0.003361	COMT	Catechol-O-methyltransferase	intron
5.0	rs316132	0.003443	GSTA4	Glutathione S-transferase A4	intron, TagSNP:GSTA4
6.0	rs1884725	0.004564	XDH	Xanthine dehydrogenase	coding-nonsynon
7.0	rs2069391	0.004830	CDK2	Cyclin-dependent kinase 2	
8.0	rs4148217	0.006167	ABCG8	ATP-binding cassette, sub-family G (WHITE), member 8 (sterolin 2)	coding-nonsynon
9.0	rs11700112	0.007423	PAK7	P21 (CDKN1A)-activated kinase 7	coding-nonsynon
10.0	rs1052536	0.007643	LIG3	Ligase III, DNA, ATP-dependent	untranslated
11.0	rs2618346	0.008033	DUSP1	Dual specificity phosphatase 1	3' UTR
12.0	rs9282564	0.008239	ABCB1	ATP-binding cassette, sub-family B (MDR/TAP), member 1	coding-nonsynon
13.0	rs53683	0.008429	GHRL	Ghrelin precursor	intron
14.0	rs2227314	0.009244	IL12A	Interleukin 12A (natural killer cell stimulatory factor 1, cytotixic lymphocyte maturation factor 1, p35)	intron
15.0	rs1801243	0.010739	ATP7B	ATPase, Cu++ transporting, beta polypeptide (Wilson disease)	coding-nonsynon
16.0	rs2953983	0.010792	POLB	Polymerase (DNA directed), beta	intron
17.0	rs4148946	0.011822	CHST3	Sarbohydrate (chondroitin 6) sulfotransferase 3	untranslated
18.0	rs7185307	0.011895	TNFRSF17	Tumor necrosis factor receptor superfamily, member 17	locus, TagSNP:TNFRSG17(BCMA)
19.0	rs699473	0.012077	SOD3	Superoxide dismutase 2, extracellular	intron
20.0	rs880324	0.012969	NFATC2	Nuclear factor of activated T-cells, cytoplasmic, calcineurin-dependent 2	intron

**Table 4:** Top SNPs ranked by univariate analysis for trial E9486.

**Rank**	**rs ID**	**pval**	**Gene Sym**	**Gene Name**	**SNP Function**
1.0	rs10018625	0.001536	TAG ERROR	TAG ERROR	unknown, TAG ERROR
2.0	rs1047643	0.001603	FDFT1	Farnesyl-diphosphate farnesyltransferase 1	coding-synon
3.0	rs20541	0.001644	IL13	Interleukin 13	coding-nonsynon
4.0	rs2108622	0.0020374	CYP4F2	Cytochrom P450, family 4, subfamily F, polypeptide 2	coding-nonsynon
5.0	rs3759259	0.002362	STYK1	Protein kinase STYK1	coding-nonsynon
6.0	rs1801133	0.003251	MTHFR	5, 10-methylenetetrahydrofolate reductase (NADPH)	coding-nonsynon
7.0	rs2069456	0.003375	CDK5	Cyclin-dependent kinase 5	intron
8.0	rs1131532	0.005650	TNFSF10	Tumor necrosis factor (ligand) superfamily, member 10	coding-synon
9.0	rs882709	0.005965	SETX	senataxin	coding-nonsynon
10.0	rs4646421	0.007847	CYP1A1	Cytochrome P450, family 1, subfamily A, polypeptide 1	intron
11.0	rs1799969	0.008221	ICAM1	Intercellular adhesion molecule 1 (CD54), human rhinovirus receptor	coding-nonsynon
12.0	rs3822430	0.009157	SRD5A1	Steroid-5-alpha-reductase, alpha polypeptide 1 (3-oxo-5 alpha-steroid delta 4-dehydrogenase alpha 1)	coding-synon
13.0	rs7903344	0.009471	CHUK	Conserved helix-loop-helix ubiquitous kinase	coding-nonsynon
14.0	rs13926	0.011531	TRAP1	TNF receptor-associated protein 1	coding-nonsynon
15.0	rs3172469	0.012006	BCL6	B-cell CCL/lymphoma 6 (zinc finger protein 51)	intron
16.0	rs215101	0.012028	ABCC1	ATP-binding cassette, sub-family C (CFTR/MRP), member 1	intron, TagSNP:ABCC1
17.0	rs2227564	0.012246	PLAU	Plasminogen activator, urokinase	coding-nonsynon
18.0	rs3096057	0.012484	CSF1	Colony stimulating factor 1 (macrophage)	Promoter
19.0	rs6474491	0.013935	STAR	Steriodogenic acute regulator	Promoter
20.0	rs2066471	0.015231	MTHRF	5, 10-methlyenetetrahydrofolate reductase (NADPH)	intron

In the univariate ranking, we did not correct for multiple comparisons; that would certainly reduce the *p*-value significance, but would not alter the rank order comparison. This approach does examine the association of each individual SNP; it is more likely that complex interactions may drive association of groups of SNPs not revealed by univariate ranking. Nevertheless, among the top ranked individual SNP variations in both trials were those associated with drug metabolism/detoxification/transport, including: cyp genes, multiple variants *of GSTA4, SLCO, UGT1, NAT2, ABCB *genes; as well as genes impacting cellular response, including: *BMP2 *(inducing myeloma apoptosis) [30], cathepsin B (inducing IL-8 dependent cellular migration and angiogenesis [31,32], *XRCC5 *(DNA repair); and genes associated with proliferative responses (*PCNA, MAPK*, cyclin kinase). The association of multiple alleles of *GSTA4 *is particularly compelling, suggesting consistency in its impact across several variant alleles. In addition, several alleles are in linkage disequilibrium, appearing as a cluster in the list – providing quality controls (as linked genes would be expected to show the same association).

**Figure 4:**
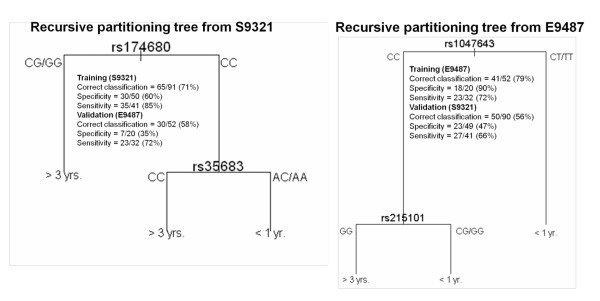
**Recursive partitioning tree from S9321 and E9486**. The classification prediction was calculated for one trial and tested on the other as validation.

The first survival separation was analyzed for clinical trial S9321, and the top 20 rank ordered SNPs are presented in Table 3 (more extended rank order presented in Table 2S of [28]). Figure 4 shows the pruned recursive partitioning tree, resulting in two SNPs with the highest classification prediction of survival groups. One SNP is in catechol methyl transferase (*COMT*) and one is in Ghrelin precursor (*GHRL*). The potential significance of these SNPs on outcomes is discussed below. The correct classification rate (survival prediction) was 71%, which dropped to 58% on validation testing with the E9486 trial. The specificity and sensitivities are also presented. The converse analysis was done (E9497 training set; S9321 validation); and the rank order of SNPs was determined (Table 4 and an expanded Table 3S in [28]). A recursive partitioning tree of two SNPs showed 79% classification on the training E9487 set, and 56% on the S9321 validation (Figure 4). The SNPs identified in this trial were farnesyl transferase (*FDFT*) and *ABCC1 *(in the family of ATP transporters). The potential significance is also provided in the *Discussion*.

As an exploratory approach, we combined the data sets from both trials, then used Fisher's exact test as a univariate screening tool to determine the association of each SNP with survival. When we treated the top 165 SNPs (univariate *p *< 0.02) as a set for short versus long PFS classification prediction using a random forest multiple sampling approach [27], we found a 79% correct classification rate. However, similar classification accuracy could be achieved with random class labels, demonstrating the potential of false positive associations in such complex data sets.

## Discussion

We have designed a novel SNP panel, containing 3404 genetic variations associated with 983 genes involved in a variety of cellular functions that could impact population variations in tumor progression and response (Table 1 and [28]). This approach is distinct from using genome-wide SNP arrays of 500,000 SNPs. The Affymetrix 500K SNP Panel is based on restriction enzyme cleavage sites and representative spacing on the chromosomes. While having significantly greater content, over 90% of the SNPs on the whole genome array are intragenic; and the chip is most often analyzed for linkage associations. The multiple comparison false positive error rate is large, and the technology considerably more expensive. Indeed, of the 3404 gene-associated SNPs on the BOAC SNP panel, only 401 are contained on the 500K SNP panel.

There are limitations to the BOAC SNP panel as well. The public and Affymetrix databases used to construct the chip content are constantly updating, so that missing elements may be noted. While we targeted SNPs in non-synonymous coding sequences or highly conserved regulatory sequences, many of the SNPs have not yet been functionally documented for effects. As such, SNP associations in the BOAC panel represent a first step in exploring the genome for clinically relevant genetic variations that will require both extensive validations as well as functional assays to confirm their effect.

We made a considerable effort to ensure that quality controls were in place. The Affymetrix platform provided a high call rate (96%) as well as very high concordance in replicate samples, even those run at different facilities. The concordance extended to 786 SNPs on the panel that were documented for the Coriell cell lines we have included [10]. All of the samples we analyzed had high quality DNA (A260/280 ratios > 1.7, and little DNA degradation). In subsequent unrelated studies, we found that even highly degraded DNA provides robust, high call rates and reproducibility (not shown); probably because the initial amplifications are across 100–150 bp of DNA. The most likely source for quality control error may come from sample misidentification or placement in multi-well plates. To control for this, we routinely incorporate randomly positioned controls and replicates.

Within the Coriell cell line panel is a distribution of racial groups. It is striking how much allelic frequencies differ in the African American vs. Caucasian racial groups. It is likely there is more refinement of allelic variations associated with more geographical based lineages [33], as racial definitions are somewhat subjective and often self reported. Importantly, as the BOAC database increases, multiple comparisons can be done with appropriate corrections for allelic variations among races. It will be important to include the full spectrum of patients as the database expands.

Disease progression, response and survival vary widely among patients. There are a number of studies that have examined variations in tumor cell chromosomal abnormalities [5] and gene expression profiles [6-8]. The evidence strongly suggests that patient outcomes are impacted by these tumor cell variations. However, patient populations show considerable germline variation that could influence the microenvironment, immune status, and drug metabolism or transport. For example, the authors (DJ, GM) have presented evidence that germline variations in *GSTP1 *show alterations in melphalan metabolism, and have been associated with different outcomes in patients receiving high dose melphalan therapies [34]. Numerous examples of variations in drug metabolism, transport, and DNA repair have been documented, with emerging associations on therapeutic outcomes.

Our approach was to provide a more global germline analysis that was driven by bioinformatic searches for potentially relevant variations in multiple genes and gene functions. This is still an exploratory approach to identify potential variations of functions that impact upon therapeutic responses and disease progression that may result in differences in survival outcomes. Rather than a linear progression of survival, we chose to examine two extreme ends of the PFS spectrum, to maximize the first steps in identifying potential functional variations. Patients were stratified by short (< 1 year) versus longer (> 3 years) PFS groups. Nevertheless, it is likely that survival is a complex endpoint resulting from both tumor progression and therapeutic failure that may impact upon multiple organ systems. Moreover, we recognized that a) tumor variation among patients may have dominant effects that are associated with survival; b) the trials we examined used multi-drug regimens, and each drug response may be impacted upon by complex genetic variations in transport, metabolism, and export; and c) sample number is still limiting statistical power. Thus, our initial approaches in this study were to determine whether germline variations had any measurable influence on survival.

We felt it was important to determine, first, if there were any true discrimination of the SNP panel in the two PFS groups, when the complete SNP profile was considered. Using a variety of methods that were tested against randomly mixed sample analysis, we found the SNP panel had true signal to discriminate the short and long progression-free survivors, although the accuracy did not reach the level of prediction that would allow clinical application. Notably, a smaller subset increased the predictive power. Significantly, no individual genetic variation provided a strong, independent prediction of survival. This likely reflects the fact that individual germline variations may impact upon response, but are not solely responsible; and it is likely that such variations are the result of complex interactions. Indeed, genetic variations in the tumor cell may play a dominant role in response and survival. Thus, patient responses are likely to involve interactions affecting multiple functions within the tumor cell as well as external factors affecting tumor progression and drug response. Nevertheless, our analysis of the SNP panel as a group suggests it is likely that germline variations impact upon patient survival and deserve further attention.

Recognizing the limited statistical power to detect single SNPs associated with PFS, we did perform a univariate analysis to rank order the SNPs that individually best discriminated the groups in the two similar phase III clinical trials. We did not correct for multiple comparisons, which would certainly reduce the *p*-value significance but would not alter the rank order comparison. This approach also assumes association for the individual SNP. It is more likely that complex multi-SNP groupings influence response. Nevertheless, among the top SNP variations in both trials were those associated with drug metabolism/detoxification/transport, including: cyp genes, multiple variants of *GSTA4*, *SLCO*, *UGT1*, *NAT2*, *ABCB *genes; as well as genes impacting cellular response, including: *BMP2 *(inducing myeloma apoptosis), cathepsin B (inducing IL-8 dependent cellular migration and angiogenesis [31,32], *XRCC5 *(DNA repair); and genes associated with proliferative responses (*PCNA*, *MAPK*, cyclin kinase). The association of multiple alleles of *GSTA4 *is particularly compelling, suggesting consistency in its impact across several variant alleles. In addition, several alleles are in linkage disequilibrium, appearing as a cluster in the list – providing quality controls (as linked genes would be expected to show the same association). Surprisingly absent from the SNP association lists are cytokines, growth factors and receptors that might be expected to cause variations in disease progression and resistance, with the exception of *IL-10*, which has been reported in previous studies [35].

While still an exploratory analysis, the paired SNPs identified by recursive partitioning in each trial have some intriguing possible connections to PFS. COMT (catechol-O-methyltransferase) metabolizes catechol drugs, and has been linked to breast cancer risk and survival [36]; GHRL has been shown to stimulate angiogenesis [37] and regulate bone formation through osteoblasts [38,39]; FDFT is the farnesyl transferase that may regulate important signaling (eg, ras) [40,41]; and ABCC is among a class of transporters that may influence multi-drug resistance [42]. It is noted that strong association in one trial was significantly reduced in the validation trial. Nevertheless, the functional impact of these genetic variations may warrant further investigation.

## Conclusion

The exploratory analyses provide some of the first attempts to use larger, targeted SNP panels to develop models of genomic variations that may influence treatment outcomes, and that may deserve further analysis of functional significance. Not surprisingly, among the most significant variations correlating with survival were genes that could be functionally categorized as pharmacologic. However, the group analysis suggests various functions may interplay in disease progression and response. It is important to consider the fact that we could not identify a small driver set of SNPs that strongly associated with survival, particularly with the limited sample size. However, we note that, as a group, germline genomic variations do have impact on event-free survival. As the Bank On A Cure data set is expanding, SNP associations are being analyzed for more specific phenotypes in response, disease complications (eg, bone disease), and adverse or toxic drug effects (eg, thrombolytic events associated with thalidomide).

Heterogeneity in tumor gene deregulation certainly contributes to variation in disease outcome. It would seem appropriate to consider combining an understanding of tumor heterogeneity (chromosomal and expression profiles) with germline variations (eg, SNP variations associated with pharmacologic functions or disease complications) that can lead to development of more individualized therapies that take into account both tumor and population variations.

## Abbreviations

BOAC: Bank On A Cure; ECOG: Eastern Cooperative Oncology Group; IRB: Institutional Review Board; MM: multiple myeloma; OR: odds ratio; PFS: progression-free survival; SNP: single nucleotide polymorphism; SVM: support vector machine; SWOG: Southwest Oncology Group; VAD: Vincristine, Adriamycin, Dexamethasone; VBMCP: Vincristine, Busulfan, Melphalan, Cyclophossphamide, Prednisone.

## Competing interests

The authors declare that they have no competing interests.

## Authors' contributions

BVN is the principal investigator of study, involved in study design and writing the manuscript. CR and MH developed genotyping assays and generated genotype data. AH, JH, and JC provided input on study design and statistical analysis. SJ provided clinical outcome data from the office of ECOG Statistical Center. MO was the clinical chair of the ECOG phase III trial. VR and PG have shared chairmanship of the ECOG Myeloma Committee and participated in clinical trial study design and clinical evaluations. BB chairs the SWOG myeloma clinical design and evaluations. BD is the Clinical Director of the Bank On A Cure project described. MK supervizes integration of laboratory efforts between the USA and the UK. VR, MS, GA, GF, RG, and RM developed statistical approaches in vector machine applications. DJ participated in SNP panel design and genotyping assembly. GM co-directs Bank On A Cure with BVN, and has been involved in study design and data evaluation.

## Pre-publication history

The pre-publication history for this paper can be accessed here:



## Supplementary Material

Additional file 1 Chromosomal distribution of BOAC SNP panel. Each SNP on the BOAC Panel is indicated in color, and indicates a broad distribution across each chromosome. Click here for file
