# Supplementary figures and images for: Genomic variation in myeloma: design, content, and initial application of the Bank On A Cure SNP Panel to detect associations with progression-free survival
Source: BMC Med. 2008 Sep 8;6:26. doi: 10.1186/1741-7015-6-26 (PMC2553089; doi:10.1186/1741-7015-6-26)

**Additional File 1: Chromosomal distribution of BOAC SNP panel**

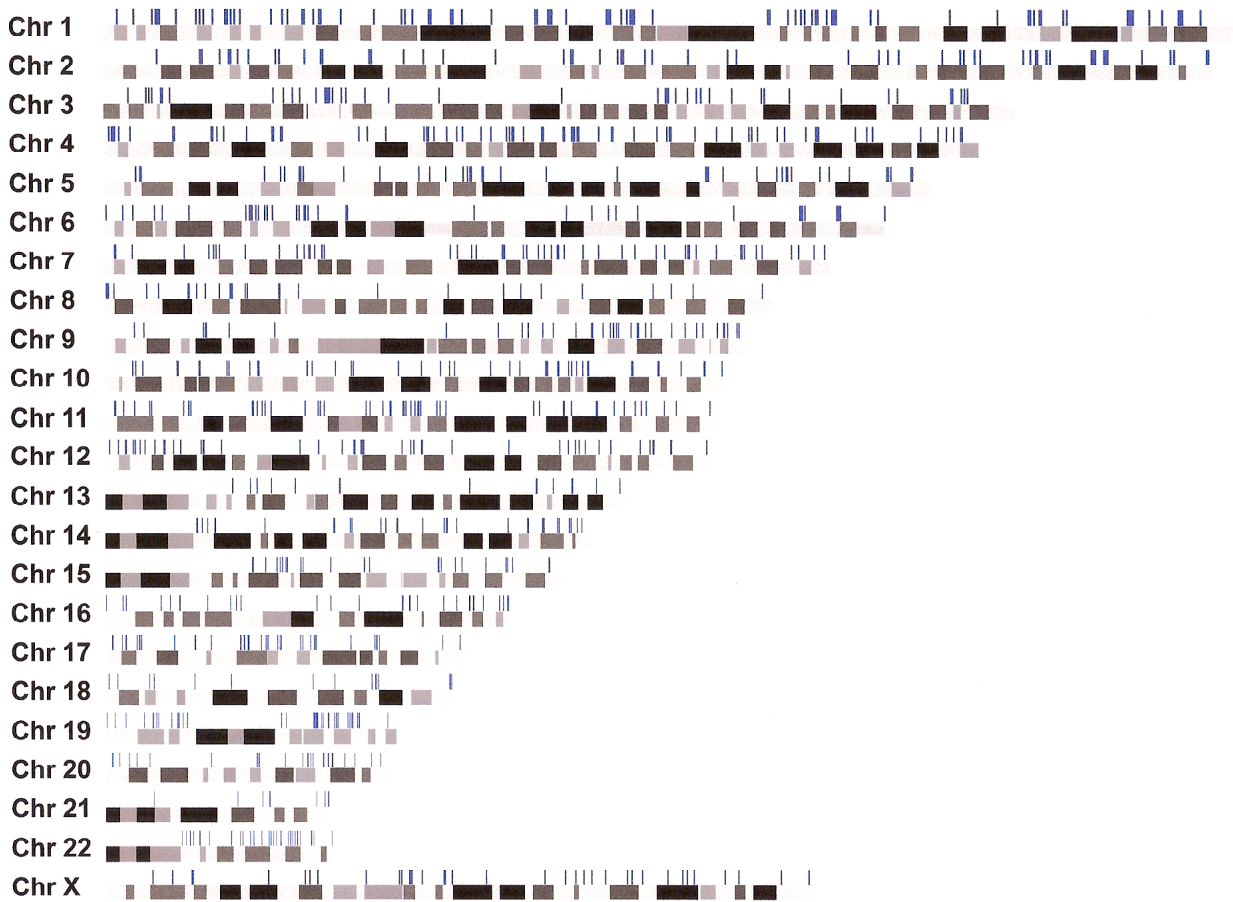

Supplement: Additional file 1 — Chromosomal distribution of BOAC SNP panel. Each SNP on the BOAC Panel is indicated in color, and indicates a broad distribution across each chromosome. [file 1741-7015-6-26-S1.pdf]
